# Dedicated Protocol for Ultrastructural Analysis of Farmed Rainbow Trout (*Oncorhynchus mykiss*) Tissues with Red Mark Syndrome: The Skin—Part One

**DOI:** 10.3390/mps7030037

**Published:** 2024-04-24

**Authors:** Diana Torge, Sara Bernardi, Giulia Ciciarelli, Guido Macchiarelli, Serena Bianchi

**Affiliations:** Department of Life, Health and Environmental Sciences, University of L’Aquila, 67100 L’Aquila, Italy; giulia.ciciarelli@graduate.univaq.it (G.C.); guido.macchiarelli@univaq.it (G.M.); serena.bianchi@univaq.it (S.B.)

**Keywords:** morphology, transmission electron microscopy, ultrastructure, red mark syndrome, rainbow trout, Midichloria-like organism

## Abstract

The present study aims to provide a specific protocol for transmission electron microscopy of a sample of skin of rainbow trout affected by red mark syndrome (RMS). The red mark syndrome is a skin disease that affects the rainbow trout (*Oncorhynchus mykiss*). The disease, probably due to the *Midichloria*-like organism infection, is not lethal, but morbidity can reach up to 60%, leading to significant economic impact associated with the downgrading of the commercial product, increased labor, and susceptibility to secondary infections. The ultrastructure analyses allowed an earlier study to identify the presence of scattered microorganisms characterized by an oval shape, mainly in the cytoplasm of the cells. The protocol developed in this study will be instrumental in visualizing the ultrastructure of the microorganism, which is probably responsible for red mark syndrome infection.

## 1. Introduction

The development of microscopical techniques, such as transmission electron microscopy (TEM), revolutionized the morphological sciences, progressively providing new levels of magnification and resolution for exploring biological and non-biological samples [1]. Since its invention in the early 1930s by Ernst Ruska and Max Knoll, TEM influenced the course of modern-day science [2], as it was the main technique that allowed the study of biological systems owing to its near-atomic-level resolution [3]. Specifically, the transmission electron microscope uses a fine electron beam determined by a high-voltage, electric current-heated tungsten filament focused by magnetic lenses [4]. This electron beam passes through an ultrathin resin section containing the investigated samples, previously post-fixed with osmium and stained with heavy metals, such as uranyl and lead citrate [4]. The different transmission of the electrons generates an image, immediately captured on a fluorescent screen and viewed by a binocular microscope. Then, the image is rapidly created by magnetic lenses and captured by a digital camera [4]. The first aim of TEM has been to image liquid samples at higher resolution. However, it was slower than with light microscopy (LM); thus, scientists have developed specific sample protocols [2,3] for observing soft and frail living matter in the inhospitable environment of an electron microscope [3]. For many scientists, TEM is a field of research, and electron microscopists have progressively improved the TEM performance and applications, leading them to new levels [2] in material sciences, physics, chemistry, and biology [2,3]. Despite its potential, the TEM application also has some limitations: the small data set of cells that can be imaged in detail and the ever-present potential for fixation and staining artifacts [5].

Regarding the advantages of this microscopic technique, TEM provides an understanding of the ultrastructure and organization of the main cellular components (cytoskeleton, membrane systems, organelles) [5]; using TEM, it was possible to clarify the structure and organization of the components of cells, and tissue [2,5] associated with their specific functional competence [6]. In the study of the cellular environment, TEM provides a black-to-white scale, two-dimensional image, highlighting, for example, the presence and distribution of heterochromatin, euchromatin, the presence of prominent nucleoli, pleomorphic nuclei, mitotic figures, and cell pleomorphism [6]. TEM also plays a crucial role in discovering and describing the ultrastructural profile of bacteria and viruses [2], such as the investigations in HIV/AIDS research and on new species of pathogens, providing complete knowledge of microorganisms and rendering their diagnosis at LM easier [5]. More recently, the high resolution of TEM also allowed the detection and visualization of the causative agent of coronavirus disease 2019 (COVID-19) [7].

Red mark syndrome (RMS) is a skin disease whose etiology has to be cleared, affecting farmed rainbow trout (Oncorhynchus mykiss) worldwide [8]. RMS is characterized by single or multiple skin lesions generally localized on the fish’s trunk, approaching market size. Even if this disease is not lethal, morbidity can reach up to 60%, leading to significant economic impact associated with the downgrading of the commercial product, treatment costs, increased labor costs, and increased susceptibility to secondary infections [9]. The causative agent of RMS remains unknown; as the disease is transmissible and sensible to antibiotic treatment, some scientific evidence suggests the involvement of a Midichloria-like organism (MLO), frequently observed in RMS lesions, which might play a pivotal role in RMS onset and progression [10]. Histopathological observations provided structural [11] and ultrastructural evidence [9], allowing the organic classification of RMS based on the severity of the lesions. Regarding RMS, the application of TEM for the identification of the causative agent for RMS allowed the visualization of specific ultrastructural details of the investigated microorganism; ultrastructural evidence revealed the presence, in samples of spleen and skin derived from rainbow trout affected by RMS, of oval or round shaped microorganisms, surrounded by a clear halo, with evidence of trilaminar cell wall structure [9]. The analyzed microorganism showed a cell wall composed of a clear outer and inner membrane (cellular envelope), indicating a trilaminar structure; this ultrastructural feature perfectly aligns with the distinct trilaminar structure frequently detected in Gram-negative bacteria [9,12]. Due to TEM’s high resolution and scientific contribution, this manuscript aims to provide an organic and complete description of TEM preparative performed on samples of skin derived from rainbow trout affected by RMS and selected for MLO detection. The presented protocol was applied to perform ultrastructural analysis on the effect of temperature on the development and resolution of RMS pathology in a previous study [9]. The presented protocol may also be applied for ultrastructural investigations on samples of skin in different physiological and pathological contexts, with multiple applications [2,3]. The mentioned study describes the presence of MLO using TEM on samples from a controlled experimental setting for the first time. As a result of applying the presented TEM preparative, these data will provide ultrastructural solid evidence that MLO is the etiological agent of RMS syndrome.

## 2. Experimental Design

**Figure 1:**
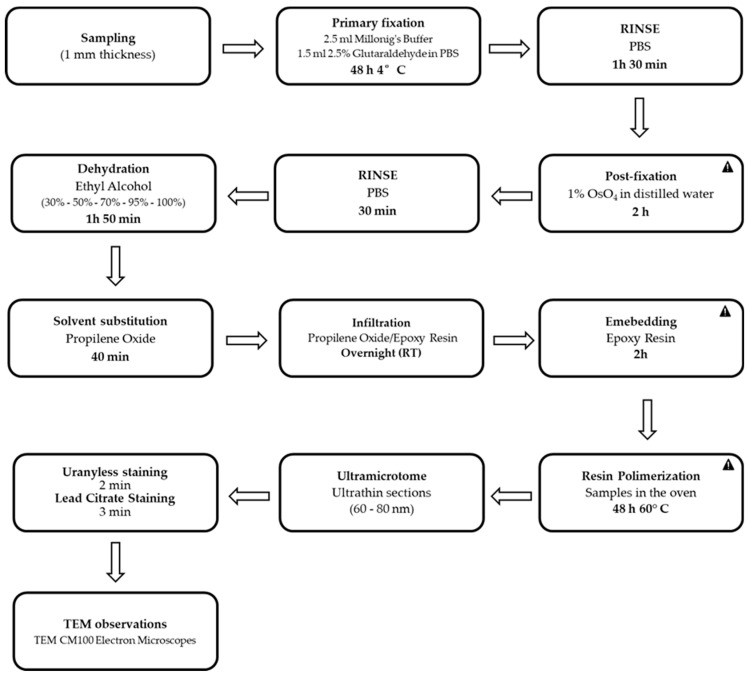
**Flow diagram of the preparative for transmission electron microscopy performed on samples of skin derived from rainbow trout affected by RMS.** 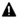: critical step of the procedure.

Samples of skin derived from rainbow trout affected by RMS were subjected to a specific preparative for TEM. Samples consisted of skin samples for MLO detection by TEM on selected cases (10 cases in total) with the most evident and severe macroscopic lesions. TEM preparative includes several stages (fixation, post-fixation, dehydration, and embedding in epoxy resin), characterized by a specific timing (Table 1, Figure 1).

### 2.1. Materials

1. Millonig’s phosphate buffer (0.1 M pH 7.4, 4) (Electron Microscopy Sciences, 1560 Industry Road, Hatfield, PA, USA, cat. no. 11582-05)
2. Glutaraldehyde EM GRADE 25% (Agar Scientific, Cambridge Road, Stansted Essex, UK, cat. no. 605390)
3. Dulbecco’s phosphate buffer (Immunological Sciences, Rome, Italy, cat. no. ISL0615-500)
4. Osmium tetroxide (OsO_4_) 4% (Electron Microscopy Sciences, 1560 Industry Road, Hatfield, PA, USA, cat. no. 19150)
5. Ethanol absolute anhydrous (Carlo Erba, Milan, Italy, cat. no. 414608)
6. EMbed-812 Embedding Kit W/DMP-30 (Electron Microscopy Sciences, 1560 Industry Road, Hatfield, PA, USA, cat. no. 14120)
7. Propylene oxide (Electron Microscopy Sciences, 1560 Industry Road, Hatfield, PA, USA, cat. no. 20401)
8. Methylene blue (Agar Scientific, Cambridge Road, Stansted Essex, UK, cat. no. 52015)
9. Borax (Merck Millipore, Burlington, MA, USA, cat. no. CA1330-43-4)
10. Azure II (Merck, Milan, Italy, cat. no. 37247-10-2)
11. Sodium hydroxide anhydrous pellets ACS-ISO for analysis (CARLO ERBA REAGENTS S.r.l., Milan, Italy, cat. no. 480507)
12. Neo-Mount^®^ (Merck Millipore, Burlington, MA, USA, cat. no. 200869).
13. Lead citrate (Agar Scientific, Cambridge Road, Stansted Essex, UK, cat. no. AGR1210)

### 2.2. Equipment

- Disposable razor blades (Electron Microscopy Sciences, 1560 Industry Road, Hatfield, PA, USA, cat. no. 71960)
- Tweezers (Electron Microscopy Sciences, 1560 Industry Road, Hatfield, PA, USA, cat. no. 78512—4DX)
- BEEM embedding capsules (Electron Microscopy Sciences, 1560 Industry Road, Hatfield, PA, USA, cat. no. 7000-B)
- Glass beakers (250–400 mL) (Sigma-Aldrich, 3050 Spruce St., Saint Louis, MO, USA, cat. no. Z231843)
- Staccup disposable beakers (120 cc) (Electron Microscopy Sciences, 1560 Industry Road, Hatfield, PA, USA, cat. no. 50-996-320)
- Magnetic stir bars (Dutscher SAS, Bernolsheim, France, cat. no. 785588)
- Magnetic stirrer (IKA-Werke GmbH & Co. KG, Staufen, Germany, cat no. 0005020000)
- Balance (Mettler Toledo, Milan, Italy, cat. no. 05361)
- Laboratory shaker (Biosan, Riga, Latvia, cat. no. BS-010130-AAI)
- Chemical fume hood (Asalair, Zetalab, Padova, Italy, cat. no. A-CARBO900)
- Oven (Memmert, Venice, Italy, cat. no. 12616987)
- Microcentrifuge (Tomos, Singapore, cat. no. 3000002)
- Parafilm (American Can Company, SPI supplies, 206 Garfield Ave West Chester, PA 19380-4512, USA, cat. no. 01851-AB)
- Glass knife maker (Leica Microsystem, Milan, Italy, cat. no. K111)
- Ultramicrotome glass knife strips (Agar Scientific, Unit 7, M11 Business Link, Parsonage Lane, Stansted, Essex CM24 8GF, UK, cat. no. AGG336)
- Microscope slides (Fischer Scientific, Rome, Italy, cat. no. 12342108)
- Coverslips (Bioptica, Milan, Italy, cat. no. 09-2040)
- Hot plate (at least 70° C) (IKA-Werke GmbH & Co. KG, 79219 Staufen, Germany, cat no. 0003582000)
- Ultramicrotome (Leica Microsystem, Milan, Italy, cat. no. Leica UC7)
- Diamon knife (Diatome Diamond Knives, Microcontrol, Milan, Italy, cat. no. TDU4530)
- Copper mesh grids (Electron Microscopy Sciences, 1560 Industry Road, Hatfield, PA, USA, cat. no. G200-Cu)
- Fine-pointed tweezers (Electron Microscopy Sciences, 1560 Industry Road, Hatfield, PA, USA, cat. no. 7801-7)
- Filter paper, hardened (Macherey-Nagel, Germany, cat. no. 431012)
- 0.22 μm filter (Fischer Scientific, Rome, Italy, cat. no. 10002261)
- Aluminum rolls (Ted Pella, Inc., Redding, CA, USA, cat. no. 43-100)
- Falcon tubes (Fischer Scientific, Rome, Italy, cat. no. 10788561)
- Microcentrifuge tubes (Fischer Scientific, Rome, Italy, cat. no. 10154671)
- Laboratory cylinder (Fischer Scientific, Rome, Italy, cat. no. 11537832)
- Glass bottles (Dutscher SAS, (Dutscher SAS, Bernolsheim, France, cat. no. 090394)
- Plastic Petri dishes (35 × 10 mm) (Biosigma, Venice, Italy, cat. no. 353001)
- Glass Petri dishes (60 × 15 mm) (Fischer Scientific, Rome, Italy, cat. no. 11740834)
- Plastic Pasteur pipettes (Merck, Milan, Italy, cat. no. BR747775)
- Glass Pasteur pipettes (Merck, Milan, Italy, cat. no. BR747725)
- Eyelash probe (Electron Microscopy Sciences, 1560 Industry Road, Hatfield, PA, USA, cat. no. 71182)
- Grid storage boxes (Electron Microscopy Sciences, 1560 Industry Road, Hatfield, PA, USA, cat. no. 71150)

## 3. Procedure

### 3.1. Preparative for Transmission Electron Microscopy

All the described procedures should be performed under a chemical fume hood.

#### 3.1.1. Preparative for TEM: Sampling

Samples consisted of skin samples for MLO detection by TEM on selected cases (10 cases in total) with the most evident and severe macroscopic lesions. Carefully sample the tissue specimen. The pieces can be small cubes, should not exceed 1 mm in thickness, and should be totally representative of the specimen.

#### 3.1.2. Preparative for TEM: Primary Fixation

Wash the skin samples derived from rainbow trout affected by RMS in a plastic Petri dish with 2.5 mL of cooled Millonig’s buffer (0.1 M pH 7.4, 4). Then, immerse the skin samples derived from rainbow trout affected by RMS in a microcentrifuge tube containing 1.5 mL of 2.5% glutaraldehyde in PBS for the primary fixation.

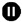
 **PAUSE STEP:** store the skin samples at 4° for 48 h (Table 1) until the start of the post-fixation.

#### 3.1.3. Preparative for TEM: Post-Fixation

After 48 h from the primary fixation, using a plastic Pasteur pipette, remove all the 2.5% glutaraldehyde from the microcentrifuge tube and transfer, using a tweezer, the skin samples derived from rainbow trout affected by RMS, in new plastic Petri dishes. In order to remove all the residues of the primary fixative, wash samples in the plastic Petri dishes with cooled PBS (0.1 M, pH 7.2) by performing three changes (30 min each), always keeping the samples on a laboratory shaker. During PBS changes, prepare OsO_4_ 1% in distilled water, according to the manufacturer’s instructions. Once the last PBS change is finished, remove all the cooled PBS from the plastic Petri dishes and transfer samples to new glass Petri dishes. At this point, using a glass Pasteur pipette, post-fix the samples with 1% OsO_4_ in distilled water for 2 h, keeping the skin samples derived from rainbow trout affected by RMS in glass Petri dishes and on the laboratory shaker.

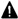
 **CRITICAL STEP:** carefully place the samples on the laboratory shaker, completely closed for 2 h, and covered with sheets of aluminum.

After the first hour of post-fixation, check the state of skin samples in the glass Petri dishes; if there are residues of the reagent, remove them and fill them again with fresh OsO_4_.

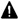
 **CRITICAL STEP:** at this stage, the skin samples derived from rainbow trout affected by RMS will be completely black; this is suggestive that post-fixation procedures are now completed.

#### 3.1.4. Preparative for TEM: Washing and Dehydration

Once the post-fixation stage is finished, wash the samples with cooled PBS by performing three changes, 10 min each, to remove all the residues of OsO_4_. Once the last PBS change is performed, start to dehydrate the samples in an ascending series of ethanol. Specifically, with plastic Pasteur pipettes, remove all the PBS from the glass dishes and fill them with ethanol 30% (one change for 10 min), ethanol 50% (one change for 10 min), and ethanol 70% (one change for 10 min).

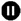
 **PAUSE STEP:** once the 70% wash is finished, remove the Petri dishes from the laboratory shaker, cover them with parafilm, and store them at 4 °C overnight.

After 24 h, continue dehydrating the samples in ethanol 95% (two changes, 10 min each) and ethanol 100% (four changes, 15 min each) by using plastic Pasteur pipettes.

#### 3.1.5. Preparative for TEM: Epoxy Resin

During the dehydration procedures, start to prepare the epoxy resin in a disposable beaker (120 cc) as follows: 20 mL of embedded resin, 16 mL of DDSA, and 8 mL of NMA.

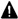
 **CRITICAL STEP:** the mix should be resuspended gently to avoid bubble formation (bubbles will impede embedding) and should be located overnight on a magnetic stirrer, in contact with a magnetic stir bar.

After 24 h, also add to this mixture 770 μL of 2,4,6-tri(dimethylaminomethyl) phenol (DMP-30) (Electron Microscopy Sciences, 1560 Industry Road, Hatfield, PA, USA).

#### 3.1.6. Preparative for TEM: The Embedding

Once the dehydration is completed by removing all the ethanol 100% from the glass Petri dishes, immerse skin samples derived from rainbow trout affected by RMS in propylene oxide (PO) for solvent substitution. At this stage, keep the Petri glass dishes open on the laboratory shaker and perform two changes of PO of 20 min by using glass Pasteur pipettes. Then, infiltrate the samples in PO/epoxy resin (1:1) overnight. After 24 h, embed these samples in the epoxy resin EMbed-812 (Electron Microscopy Sciences, 1560 Industry Road, Hatfield, PA, USA).

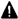
 **CRITICAL STEP:** keep the glass Petri dishes containing the samples closed for 1 h on the laboratory shaker.

After 1 h, remove all the epoxy resin from glass Petri dishes and add further resin to them for infiltration. Keep the dishes closed on the laboratory shaker for 1 h.

Meanwhile, prepare beam embedding capsules. Finally, remove all the resin from the dishes and transfer the sample to beam embedding capsules, filling them with epoxy resin. Using forceps, place a small, elongated piece of paper (rectangular) marked with a pencil to identify the tissue/block inside the outer, upper part of each capsule.

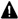
 **CRITICAL STEP:** keep the embedded samples on the laboratory shaker for 1 h and then put them at 60° in the oven for 48 h.

After 48 h, remove the skin-embedded samples kept at 60° for 48 h from the oven (Table 1).

#### 3.1.7. Preparative for TEM: The Ultramicrotome

Embedded samples, removed from the oven at 60°, are sectioned using a Reichert–Jung Ultracut E ultramicrotome to obtain semithin sections (1 μm thick).

#### 3.1.8. Preparative for TEM: Semithin Sections

Specifically, place skin samples derived from rainbow trout affected by RMS in the ultramicrotome by using disposable razor blades to trim the face of these samples like they resemble a trapezoid. Remove as much epoxy resin as possible. By using a glass knife, section the sample until the tissue is revealed, and then cut some semithin sections. At this stage, by using a plastic Pasteur pipette, place some drops of distilled water on a slide. Then, by using the eyelash probe, collect some semithin sections (one for each drop). Place these slides on a hot plate (70°–90°), allowing the drops to evaporate. Stain semithin sections of the skin of rainbow trout affected by RMS with methylene blue.

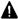
 **CRITICAL STEP:** filter the methylene blue solution, previously prepared, with a syringe and a 0.22 μm filter.

Use a plastic Pasteur pipette to place some drops of this stain on the semithin sections located on the slides and keep on a hot plate (70°–90°) to allow the drops to evaporate. During this step, leave the slide on the hot plate for a further minute to ensure that semithin sections adhere to the slide and are flattened out. Rapidly wash the samples with distilled water and keep them on the hot plate again to remove any drops of stain and distilled water. Then, examine the obtained semithin sections using LM (Zeiss Axioskop, Zeiss, Oberkochen, Germany) and photograph them by using a digital camera (Leica DFC230, Wetzlar, Germany). The samples should stain to varying degrees of blue and should be mounted with a new rapid mounting medium for microscopy Neo-Mount^®^.

#### 3.1.9. Preparative for TEM: Ultrathin Sections

Mount the diamond knife in the holder of the ultramicrotome, and fill water through with filtered double-distilled water. Ensure the knife edge is wet. If necessary, use the eyelash probe (eyelash mounted to the end of an applicator stick) to brush water onto the edge. Cut ultrathin sections (60–80 nm) with the diamond knife and mount them on copper grids. Place grids in a slotted storage box.

#### 3.1.10. Preparative for TEM: Uranyless and Lead Citrate Staining

Stain the copper grids containing the ultrathin sections of skin samples with Uranyless and lead citrate (SIC, Rome, Italy). Specifically, the filtered paper should be placed into a box to isolate the samples. Place also the top of a glass Petri dish into the box and cover it with parafilm. By using a fine-pointed tweezer, place the copper grids containing the ultrathin sections on the paper and cover them with a further top of a glass Petri dish. Meanwhile, place some drops of Uranyless on the glass and place the copper grids on the drops for 2 min. After this, wash the copper grids with distilled water. Then, place the copper grids on filtered paper to allow the residues of the drops to evaporate. After this step, cover the stained copper grids with the top of a Petri glass, and by using glass pipettes, put some drops of lead citrate on the glass. Then, also place the copper grids on the drops for 3 min. Finally, wash the copper grids with distilled water. Then, place the copper grids on filtered paper again to allow the residues of the drops to evaporate. Once finished, store the stained copper grids containing the ultrathin sections of skin derived from rainbow trout affected by RMS in a grid storage box.

#### 3.1.11. Preparative for TEM: TEM Observations

Examine and photograph the ultrathin sections of skin samples derived from rainbow trout affected by RMS by using Philips TEM CM100 Electron Microscopes, operating at 80 kV.

## 4. Results and Discussion

**Figure 2:**
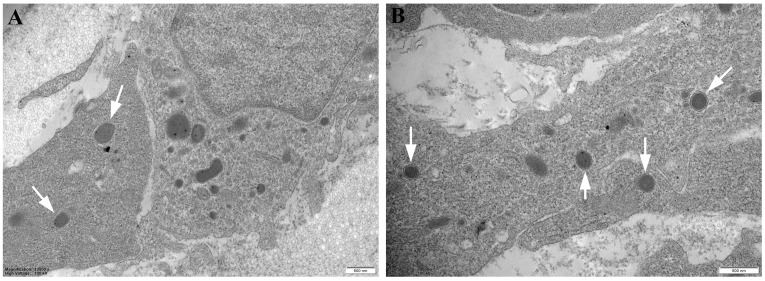
**Representative TEM micrograph of Skin of RMS Rainbow Trout (Ultrathin section: 80 nm)** (Courtesy: Orioles, M.; Galeotti, M.; Saccà, E.; Bulfoni, M.; Corazzin, M.; Bianchi, S.; Torge, D.; Macchiarelli, G.; Magi, G.E.; Schmidt, J.G. Effect of Temperature on Transfer of Midichloria-like Organism and Development of Red Mark Syndrome in Rainbow Trout (*Oncorynchus mykiss*). Aquaculture 2022 [9]). (**A**) TEM micrograph of round shaped microorganisms, with trilaminar cell wall structure in the skin. Magnification: 13,500 X (Bar: 500 nm). (**B**) Multiple round shaped microorganisms in the skin of RMS Rainbow Trout. Magnification: 19,000 X (Bar: 500 nm). Arrow: microorganism.

The application of the described protocol provided ultrastructural evidence on the presence of specific microorganisms in skin samples derived from rainbow trout affected by RMS. Specifically, TEM detected the presence of scattered microorganisms, probably inside the cytoplasm of the cells (Figure 2). At the same time, TEM was also fundamental in describing the ultrastructural profile of the examined pathogen. Ultrastructural analysis on the effect of temperature on the development and resolution of RMS pathology, conducted using TEM [9], described the presence of MLO on samples from a controlled experimental setting for the first time (Figure 2). Furthermore, the examined microorganisms were round-shaped, with evidence of trilaminar cell wall structure (Figure 2) [9]. Therefore, the presented protocol allowed us to hit an important goal of TEM analysis: to provide a spatial and morphological description of the investigated microorganism inside the skin of rainbow trout affected by RMS. RMS syndrome is associated with water temperatures below 16 °C, can still be present at 2 °C, and the healing time seems to be associated with the water temperature [9]. Recent scientific evidence suggested that if it is possible to raise the water temperature, it will be possible to accelerate the development of the disease and to modify the severity of the pathology [9]. RMS disease seems transmissible, and current scientific evidence individuated a bacterial infection as the likely etiology [13]. Specifically, MLO is a Rickettsia-like organism (RLO) belonging to the Midichloriaceae family and a member of the order Rickettsiales [13]. This microorganism was often visualized in splenic impression smears, like morula-like structures, typical of Rickettsia-like organisms, in the cytoplasm of splenic macrophages of RMS-affected rainbow trouts [13].

These data, as a result of the application of the presented TEM preparative, provided significant ultrastructural evidence that MLO is the etiological agent of RMS syndrome. Moreover, evidence derived from immunohistochemistry reported positive staining of MLO-related antigens in the internal organs of rainbow trout affected by RMS [8], confirming the potential role of this specific class of microorganisms in the onset and progression of RMS disease. Also, phylogenetic studies reported the presence of RLOs in trout with RMS by shedding light on a new clade–family within Rickettsiales (Alphaproteobacteria), the Midichloriaceae, including several bacterial symbionts and the bacteria Midichloria mitochondrii, a symbiont of the hard tick Ixodes ricinus [13]. Instead, clinical, histopathological [14], and molecular biology analysis also contributed to the diagnosis of RMS [9], providing morpho-functional evidence on the dynamics underlying RMS syndrome [15]. From a molecular point of view, several studies documented the supposition of a causative role of MLO in RMS, supported by the association of higher numbers of nucleic acid sequences associated with the severity of the lesions [9]. RMS diagnosis is mainly based on clinical features and histopathology, complemented by molecular evidence, generally derived from PCR for MLO DNA [13]. As confirmed by recent molecular studies, concentrations of MLO DNA were detected by both quantitative polymerase chain reaction (qPCR) and digital droplet polymerase chain reaction (ddPCR) techniques in samples of skin and spleen derived from rainbow trout affected by RMS [9].

**Figure 3:**
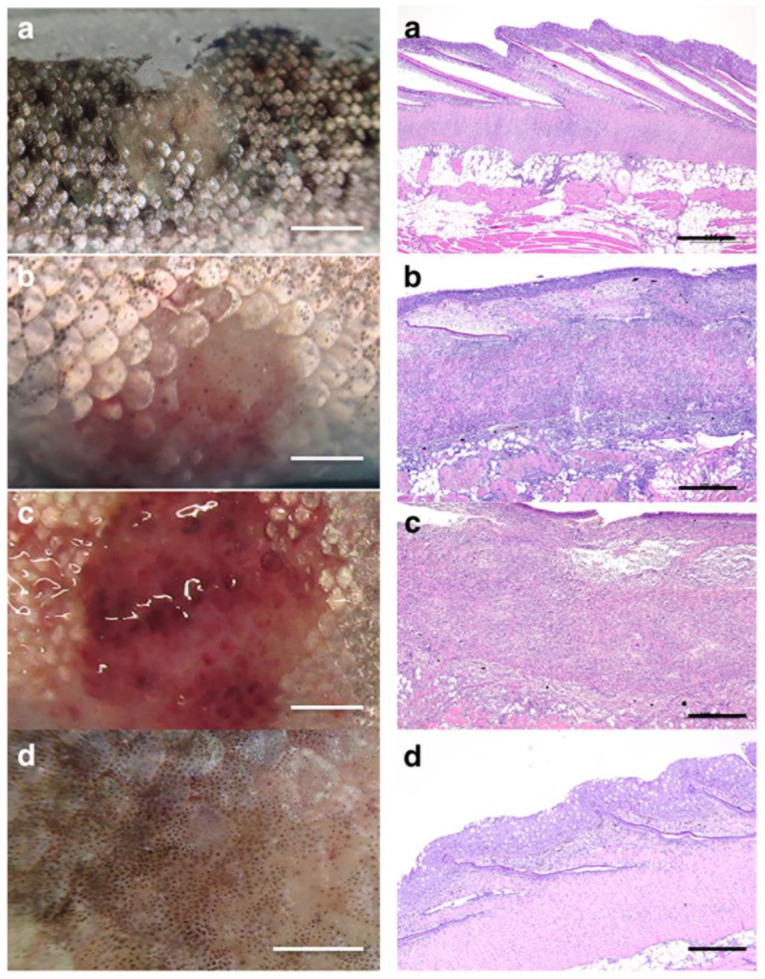
**Macroscopic and microscopic examinations (LM) of mild (a), moderate (b), severe (c), and healing (d) RMS skin lesions (white Bar: 0.5 cm, black Bar: 500 μm).** (Courtesy: Orioles, M.; Galeotti, M.; Saccà, E.; Bulfoni, M.; Corazzin, M.; Bianchi, S.; Torge, D.; Macchiarelli, G.; Magi, G.E.; Schmidt, J.G. Effect of Temperature on Transfer of Midichloria-like Organism and Development of Red Mark Syndrome in Rainbow Trout (Oncorynchus mykiss). Aquaculture 2022 [9]). Left side images (**a**–**d**): macroscopical examination. Right side images (**a**–**d**): microscopical examination (LM).

Furthermore, histopathological observations provided structural evidence, which allows an organic classification of RMS based on the severity of the lesions [11]. As reported in the literature, using LM, RMS lesions were classified from a macroscopic and microscopic point of view [11]. RMS may be characterized by mild skin lesions [9], with prevalent lymphocytic inflammatory infiltrate, limited to the dermis spongiosum, sometimes by moderate lesions, with necrosis and a higher level of lymphocytic inflammation affecting dermis spongiosum (Figure 3) [9]. Instead, severe lesions of RMS are macroscopically characterized by marked redness and hemorrhages while microscopically by a massive lymphocytic infiltration throughout the dermis and visible necrosis (Figure 3) [9]. Finally, there is no lymphocytic inflammatory infiltrate in the healing/regenerative stages (Figure 3) [9]. Instead, there is little evidence regarding the ultrastructural features associated with the progression of RMS syndrome in different tissues derived from affected rainbow trout. The combination of ultrastructural and molecular evidence will be helpful to afford crucial insights into the pathogenesis of RMS [13,16], achieving a broader comprehension of the mechanisms driving this condition, probably not restricted to the skin tissue, but involving different targets, from spleen to kidney [9,13].

This study presents a detailed TEM preparative for skin samples derived from rainbow trout affected by RMS syndrome. These procedures led to the detection of the causative agent of this disease in the investigated samples, as well as providing an ultrastructural overview of the investigated MLO. Because the pathogenesis of RMS in rainbow trout is a subject of ongoing debate among the scientific community [10,17], and previous studies have faced elucidating this issue [18,19], mainly shedding light on skin tissue, but sometimes failing to examine other organs, like spleen, and kidney [13], to advance our understanding of the pathogenic mechanisms underlying RMS, this protocol provides a detailed description of the main procedures, requested for TEM analysis, performed on samples of skin-derived from rainbow trout, affected by RMS disease. The ultrastructural observations provided by applying this TEM preparative may be informative of the shape and distribution of the investigated microorganism within the target tissue.

Furthermore, the high resolution of the electron microscopy allows the visualization of specific ultrastructural details of the microorganism, for example, regarding the ultrastructure of the cell wall, by examining the morphology of the cellular envelope. Moreover, TEM observations derived from applying the presented protocol were useful in describing the so-called trilaminar structure frequently observed in Gram-negative bacteria [9].

As suggested by previous studies, these microorganisms were generally detected in splenic erythrocytes/macrophages and skin macrophages/fibroblasts [9]. From a methodological point of view, this protocol adapts the standard preparative, generally applied for TEM analysis, to a new and different class of samples, skin derived from rainbow trout affected by RMS. The histological and ultrastructural evaluation of this class of samples made it possible to describe the specific pattern of RMS lesions, easily comparable to evidence already reported by several authors [20]. Furthermore, one ultrastructural study performed in RMS trout did not detect the presence of intralesional microorganisms [21]. In contrast, a recent TEM study reported the presence of intracellular bacteria characterized by ultrastructural features, similar to bacteria belonging to Rickettsiaceae and Midichloriaceae families, order Rickettsiales, as regards the shape, dimension, cell wall organization, and subcellular localization [9,12].

The ultrastructural evidence derived from this study, combined with molecular data obtained by second-step PCR, supported the hypothesis that TEM detected microorganisms belonging to the order Rickettsiales [9,12] by confirming the role of a Rickettsiales-like organism in the etiology of RMS [13]. For these reasons, applying the described protocol will be useful in providing evidence regarding the localization of the investigated microorganism in the selected tissues. With this aim, the proposed protocol and the associated high-resolution microscopic technique will lead to a spatial and ultrastructural profile of the investigated microorganism, adding more important details to the onset, progression, pathogenesis, and probably diagnostics of RMS. Finally, for the first time, the application of this protocol will shed light on the illustration through TEM of the presence of MLOs associated with RMS in an experimental setting.

## 5. Conclusions

This is a complete, “step-by-step” protocol for TEM analysis performed on samples of skin derived from rainbow trout affected by RMS syndrome. This protocol adapts the standard preparative, generally applied for TEM analysis, to a new and different class of samples, skin derived from rainbow trout affected by RMS, with the aim to discover the causative agent of this disease and describe its ultrastructural features for the first time. This protocol might also be applied to different types of samples derived from rainbow trout affected by RMS, with the aim of providing an ultrastructural profile of MLO frequently observed from RMS lesions. At the same time, the presented protocol may also be applied for ultrastructural investigations on samples of skin in different physiological or pathological conditions, with interesting implications. Our method enabled a thorough exploration of the ultrastructural dynamics that underlie the pathogenesis of RMS in the skin of rainbow trout. Further ultrastructural examinations are needed and might contribute to a better understanding of the onset and progression of RMS in the skin of rainbow trout in freshwater aquaculture.

## 6. Reagent Setup

### 6.1. Primary Fixation Reagent: 2.5% Glutaraldehyde in PBS

Prepare 2.5% glutaraldehyde in PBS: by using a plastic pipette and a falcon tube (50 mL), dissolve 5 mL of 25% glutaraldehyde in 45 mL of cooled PBS.

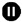
 **PAUSE STEP:** then, carefully store the falcon tube containing 2.5% glutaraldehyde at 4 °C until the sampling and fixation procedures.

### 6.2. Dehydrating Solutions: Ethanol 30–100%

- Prepare ethanol 30% in distilled water: by using a plastic Pasteur pipette and falcon tubes (50 mL), dissolve 15 mL of ethanol absolute anhydrous in 35 mL of distilled water. Carefully store the falcon tubes at 4° C until dehydration.
- Prepare ethanol 50% in distilled water: by using a plastic Pasteur pipette and falcon tubes (50 mL), dissolve 25 mL of ethanol absolute anhydrous in 25 mL of distilled water. Carefully store the falcon tubes at 4° C until dehydration.
- Prepare ethanol 70% in distilled water: by using a plastic Pasteur pipette and falcon tubes (50 mL), dissolve 35 mL of ethanol absolute anhydrous in 15 mL of distilled water. Carefully store the falcon tubes at 4° C until dehydration.
- Prepare ethanol 95% in distilled water: by using a plastic Pasteur pipette and falcon tube (50 mL), dissolve 47.5 mL of ethanol absolute anhydrous in 2.5 mL of distilled water. Carefully store the falcon tubes at 4° C until dehydration.
- Prepare ethanol 100%: by using a plastic Pasteur pipette, place in a falcon tube (50 mL) a total volume of 50 mL of ethanol absolute anhydrous. Then, carefully store the falcon tubes at 4° C until dehydration.

### 6.3. Post-Fixation Reagents: OsO_4_ 1% in Distilled Water

Prepare OsO_4_ 1% in distilled water for a total volume of 10 mL of solution in a glass bottle. Specifically, by using a glass Pasteur pipette, dissolve 2 mL of OsO_4_ 4% in 8 mL of distilled water.

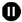
 **PAUSE STEP:** then, carefully close and cover the glass bottle containing OsO_4_ 1% in distilled water with aluminum sheets, and store it at 4° C until the post-fixation.

### 6.4. LM Staining: Methylene Blue

Regarding the methylene blue solution, prepare three falcon tubes (50 mL), filling two of them with 25 mL of distilled water each and leaving the third tube completely empty. In the first falcon tube with distilled water, dissolve 0.25 gr of Borax and 0.25 gr of methylene Blue. In the second falcon tube, previously filled with 25 mL of distilled water, dissolve 0.25 gr of azure blue. Vortex all the falcon tubes and transfer the 25 mL of the first tube to the second one for a total volume of 50 mL. Filter this total volume in a new falcon tube by using a 0.2 μm, and vortex the falcon tube. After this step, name the falcon tube, completely cover the solution with an aluminum sheet, and store it at 4° C.

### 6.5. TEM Staining: Lead Citrate

- Firstly, prepare sodium hydroxide Na(OH) 1N: dissolve in a glass bottle 4 gr of Na(OH) in 10 mL of cold distilled water. Mix the solution until it is completely dissolved, and cover it with parafilm.

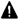
 **CRITICAL STEP:** at this stage, the mixture will be hot: mix it until it is completely clear and ready for use.

- Prepare lead citrate 0.3% in distilled water: place 30 mL of distilled water, collected in a becker, on a magnetic stirrer and wait until it boils. After this step, transfer all 30 mL of distilled water to a laboratory cylinder and store it at 4° C. Once the solution is completely cold, dissolve in it 1.33 gr of lead nitrate PB(NO_3_)_2_ and 1.76 gr of trisodium citrate dihydrate Na_3_(C_6_H_5_O_7_) 2H_2_O, with 8 mL of sodium hydroxide Na(OH) 1N, previously prepared, also adding distilled water to reach a total volume of 50 mL.

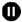
 **PAUSE STEP:** then, store the prepared solution at 4°C until the start of the lead citrate staining.

## Figures and Tables

**Table 1:** Preparative for transmission electron microscopy (fixation, post-fixation, dehydration, and embedding in epoxy resin).

PROCEDURE	REAGENTS	TIME
**FIXATION**	Millonig’s Buffer (0.1 M ph 7.4, 4) and Glutaraldehyde 2.5% in PBS	48 h at + 4 °C
**RINSE**	PBS	30 min (3 changes)
**POST - FIXATION**	Osmium Tetroxide 1% in distilled water	2 h
**RINSE**	PBS	10 min (3 changes)
**DEHYDRATION**	Ethyl Alcohol 30% Ethyl Alcohol 50% Ethyl Alcohol 70% Ethyl Alcohol 95% Ethyl Alcohol 100%	10 min 10 min 10 min 10 min (2 changes) 15 min (4 changes)
**SOLVENT SUBSTITUTION**	Propylene Oxide	20 min (2 changes)
**INFILTRATION**	Propilene Oxide:Epoxy Resin (50/50)	Overnight at RT
**EMBEDDING**	Epoxy Resin	2 h
**EMBEDDING AND RESIN POLYMERIZATION**	Epoxy Resin	48 h at 60 °C

## Data Availability

Data will be available upon kind request from the corresponding author, D.T.

## Abbreviations

COVID-19	coronavirus disease 2019
ddPCR	digital droplet PCR
DDSA	dodecenylsuccinic anhydride, specially distilled
DMP-30	2,4,6-tri(dimethylaminomethyl) phenol
LM	light microscopy
MLO	*Midichloria*-like organism
Na_3_(C_6_H_5_O_7_)2 H_2_O	trisodium citrate dihydrate
NMA	methyl-5-norborene-2,3-dicarboxylic anhydride
Na(OH)	sodium hydroxide
OsO_4_	osmium tetroxide
PB(NO_3_)_2_	lead nitrate
PO	propylene oxide
qPCR	quantitative PCR
RLO	*Rickettsia*-like organism
TEM	transmission electron microscopy
